# Evaluation of Pregnancy, Younger Age, and Old Age as Independent Risk Factors for Poor Hospitalization Outcomes in Influenza A (H1N1)pdm09 Virus a Decade After the Pandemic

**DOI:** 10.7759/cureus.11762

**Published:** 2020-11-28

**Authors:** Sathyamurthy P, Senthil Kumar Dhandapani N

**Affiliations:** 1 Internal Medicine, Sri Ramachandra Institue of Higher Education and Research, Chennai, IND; 2 Internal Medicine, Sri Ramachandra Institute of Higher Education and Research, Chennai, IND

**Keywords:** influenza, elderly, pregnant women, mortality, h1n1, hospital outcomes, symptoms, comorbidities, superinfections, risk factors

## Abstract

Introduction

The influenza A (H1N1)pdm09 virus infection was first reported in Mexico in 2009 and quickly became the first flu pandemic of the 21st century. Statistics show that the prevalence of H1N1 infection was higher among young adults during the pandemic while the elderly were at more risk of death. However; many studies have shown a gradual change over the years, with attack rates increasing in older adults as compared to young adults. The other significant vulnerable group for this infection seems to be pregnant women. Over the years, many authors have found that pregnancy may not be a significant risk factor for increased hospitalization and poorer outcomes. This study aims to perform a comparative analysis and thereby assess pregnancy, younger age, and old age as independent risk factors for poor hospitalization outcomes.

Materials and methods

The hospital records of all patients with H1N1 infection admitted between January 1, 2018, to December 31, 2018, were screened. The patients included in the study were young adults (18-31 years), pregnant women, and the elderly (≥65 years). Comparative analysis was done between them. Nominal variables were compared using the chi-square test.

Results

A total of 379 patients were admitted to our hospital with H1N1 infection from January 1, 2018, to December 31, 2018. There were 75 elderly (19.7%), 224 (59%) middle-aged adults, 55 (14.5%) young adults, and 25 (6.5%) pregnant women. Fever (90%, 84%, and 96%) and cough with expectoration (72%, 67.3%, and 40%) were the most prevalent symptoms. The elderly reported more dyspnoea (28% vs. 5.5%, 4 %). Diabetes mellitus was found in 73.3 % of the elderly, 3.6% of the young adults, and 12% of pregnant women. Hypertension was present in 45% of the elderly, 1.8% of young adults, and 4% of pregnant women. Coronary artery disease was seen in 22.7% of the elderly and 1.8% of young adults. Chronic kidney disease (5.3%) and chronic obstructive pulmonary disease (13.3%) were seen only in the elderly group. Relative lymphopenia was prevalent in all groups and was more in pregnant women (76% vs. 61.8% and 41.8%) as compared to other groups. Serum creatinine was elevated in 38% of the elderly, 2% of young adults, and 0% of pregnant women. Abnormal chest radiograph was reported for 48% of the elderly, 30.9% of young adults, and 12% of pregnant women. Twenty-six point seven percent (26.7%) of the elderly needed more than a weeks' stay as compared to 7.3% of young adults and 20% of pregnant women. Thirty-two percent (32%) of the elderly required intensive care as compared to 1.5% of young adults and none of the pregnant women. More of the elderly (26.7%) required ventilator support than other groups (7.3% and 4%). About 25.3% of the elderly had a superinfection. Eight percent (8%) of the elderly died in the study while none died in the other groups.

Conclusion

Age representation and poor hospitalization outcomes due to H1N1 seem to have shifted from young adults to older age groups. The elderly are at more risk for a prolonged stay, intensive care, ventilator support, and death as compared to young adults and pregnant women. Pregnancy may not be associated with poor hospitalization outcomes for H1N1 as has been earlier thought.

## Introduction

The influenza A (H1N1)pdm09 virus infection was first reported in Mexico in 2009, and quickly became the first flu pandemic of the 21st century, causing infections in more than 214 countries [1]. It has now been a decade since the virus was first reported. The clinical characteristics and hospitalization outcomes of this strain of influenza have been more or less similar to another influenza A subtype strains, except for a few reported differences such as younger age representation, atypical presentation, and association of lymphopenia [2]. Since 2010, this virus has been reported to have caused waves of seasonal epidemics throughout the world. Fever and cough have been the most commonly reported symptoms [1,3-4] while dyspnea [5] and extensive radiological involvement [6] have been shown to be associated with poor outcomes. Elderly people are likely to manifest less systemic symptoms and more atypical symptoms, such as confusion and disorientation [7]. The presence of comorbidities such as diabetes mellitus (DM), cardiovascular diseases, chronic respiratory diseases, and chronic kidney disease (CKD), has been shown to cause an increase in hospital morbidity and mortality [6,8-11]. The age-specific attack rate, complications, morbidity, and mortality have also varied over the multiple waves since the pandemic first began [8,12-13].

Statistics show that the prevalence of H1N1 infection was higher among younger adults than among the elderly during the pandemic [3]. In fact, about 60% of cases affected individuals between 10 and 30 years of age while the elderly represented less than 1% of cases [1,3]. The proportion of those who died consisted more of younger and middle-aged adults than the elderly [14]. The probable reason for the lesser attack rates of infection among the elderly, in particular, those who were born before 1950, is due to the prevalence of cross-reactive antibodies to H1N1, potentially as high as 34% in them [7]. Though the attack rates were less, the hospitalization outcomes have been poorer among the elderly compared to young adults during the initial wave of the pandemic and in subsequent seasonal epidemics throughout the world [3,7,15]. However, many studies have shown a gradual change over the years with attack rates increasing in older adults as compared to younger adults [8,13,16]. Whether young adults who were more vulnerable to increased attack rates and deaths during the pandemic are still so is unclear. The other significant vulnerable group for this infection seems to be pregnant women [3,9-10]. In this group, the physiological changes associated with pregnancy and the deviation of the immune system from cellular to humoral has been postulated to be the reason for the increased rate of hospitalization and mortality [17]. In contrast, many authors have found that pregnancy may not be a significant risk factor for increased hospitalization and poorer outcomes [6,15,18].

In short, it has been shown that young adults are more prone to H1N1 infection and that the elderly are more prone to poor hospitalization outcomes while pregnant women are unique in that they are young, yet have a high risk for severe disease and complications. Since there is an age shift, a change in attack rates, changes in epidemiology, and conflicting evidence, the above statement needs to be reconsidered. There could be significant variation among the clinical and laboratory spectrum between these three groups as well [3,7]. This study aims to perform a comparative analysis between young adults, elderly individuals, and pregnant women hospitalized with H1N1, and thereby assess pregnancy, younger and old age as independent risk factors for poor hospitalization outcomes a decade after the pandemic was first declared.

## Materials and methods

The study was approved by the institutional ethical committee. The study was done in patients admitted at our center between January 1, 2018, and December 31, 2018, with confirmed H1N1 infection. H1N1 infection was confirmed by pharyngeal swab real-time polymerase chain reaction (RT-PCR) in suspected cases. The study included all RT-PCR positive adult patients (≥ 18years of age) admitted during the study period. Pediatric patients (< 18 years) were excluded from the study.

Patients were divided into four categories: young adults (defined as patients in the age group 18-31 years for our study purpose, excluding pregnant women); middle-aged adults (32-64 years, excluding pregnant women); pregnant women of all ages; and the elderly (defined as patients with age ≥65 years for our study). The above categorization was made to assess young adulthood and pregnancy as independent risk factors. Since this is a comparative analysis between three groups as mentioned in the introduction of the study, middle-aged adults were excluded from the analysis.

Data pertaining to the study population were retrospectively extracted from the hospital records using a standardized proforma. Extracted data were categorized into clinical features, comorbidities, laboratory features, and hospitalization outcomes. Data were entered into Microsoft Excel (Microsoft Corporation, Redmond, WA) and analyzed using the Statistical Package for the Social Services (SPSS) software version 26 (IBM Corp., Armonk, NY) at Chennai, India. Age distribution was expressed in terms of median and interquartile range. Pie charts were used to express the ages and categorical distributions. Other variables were described in percentages (%) for each group. Comparative analysis was performed between the three groups for clinical features, comorbidities, laboratory features, and hospitalization outcomes. Categorical variables were compared using Pearson’s chi-square test. An alpha value of .005 was considered statistically significant.

## Results

**Figure 1:**
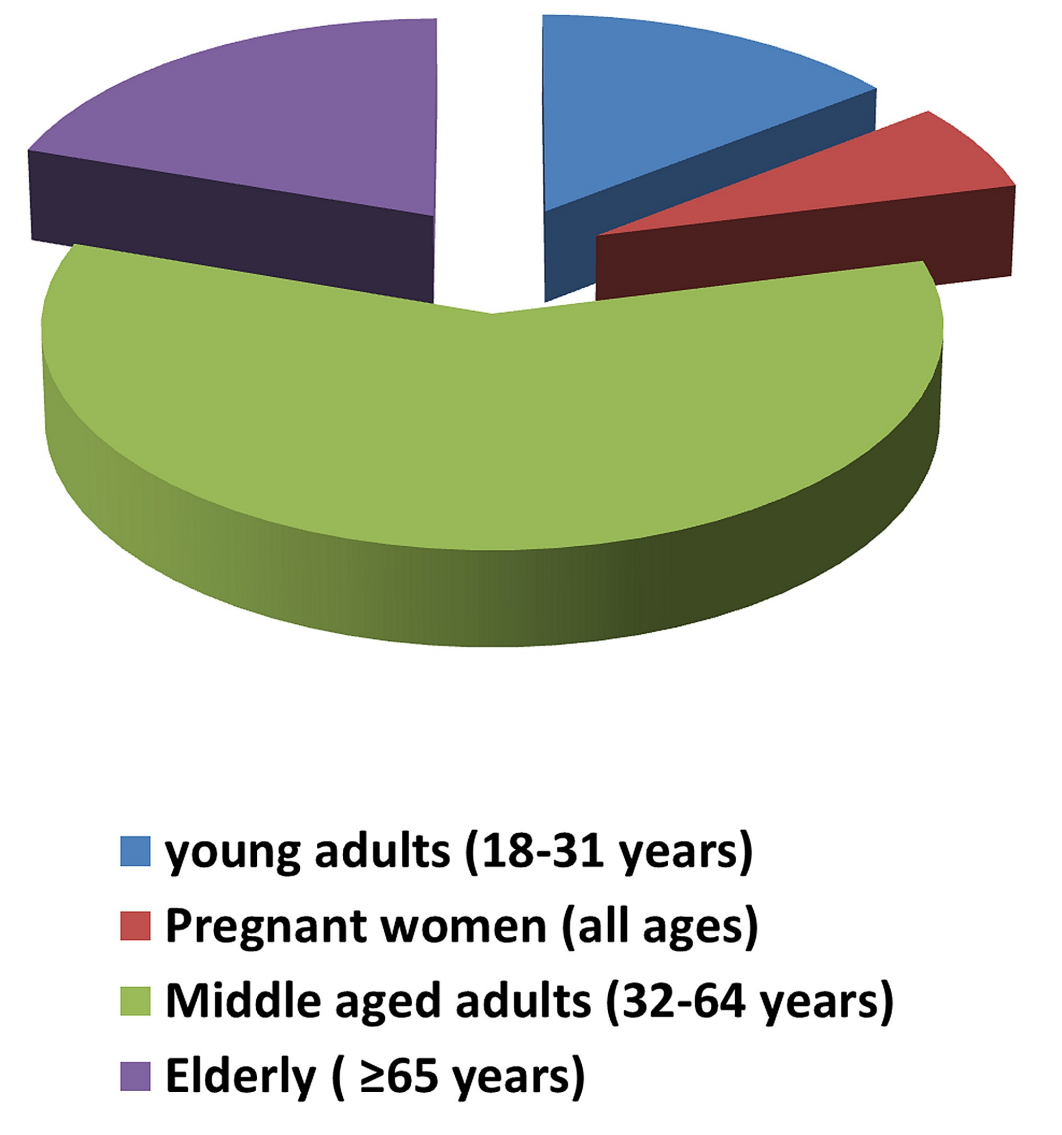
Representation of various categories of individuals admitted to the hospital with H1N1 infection in 2018

**Figure 2:**
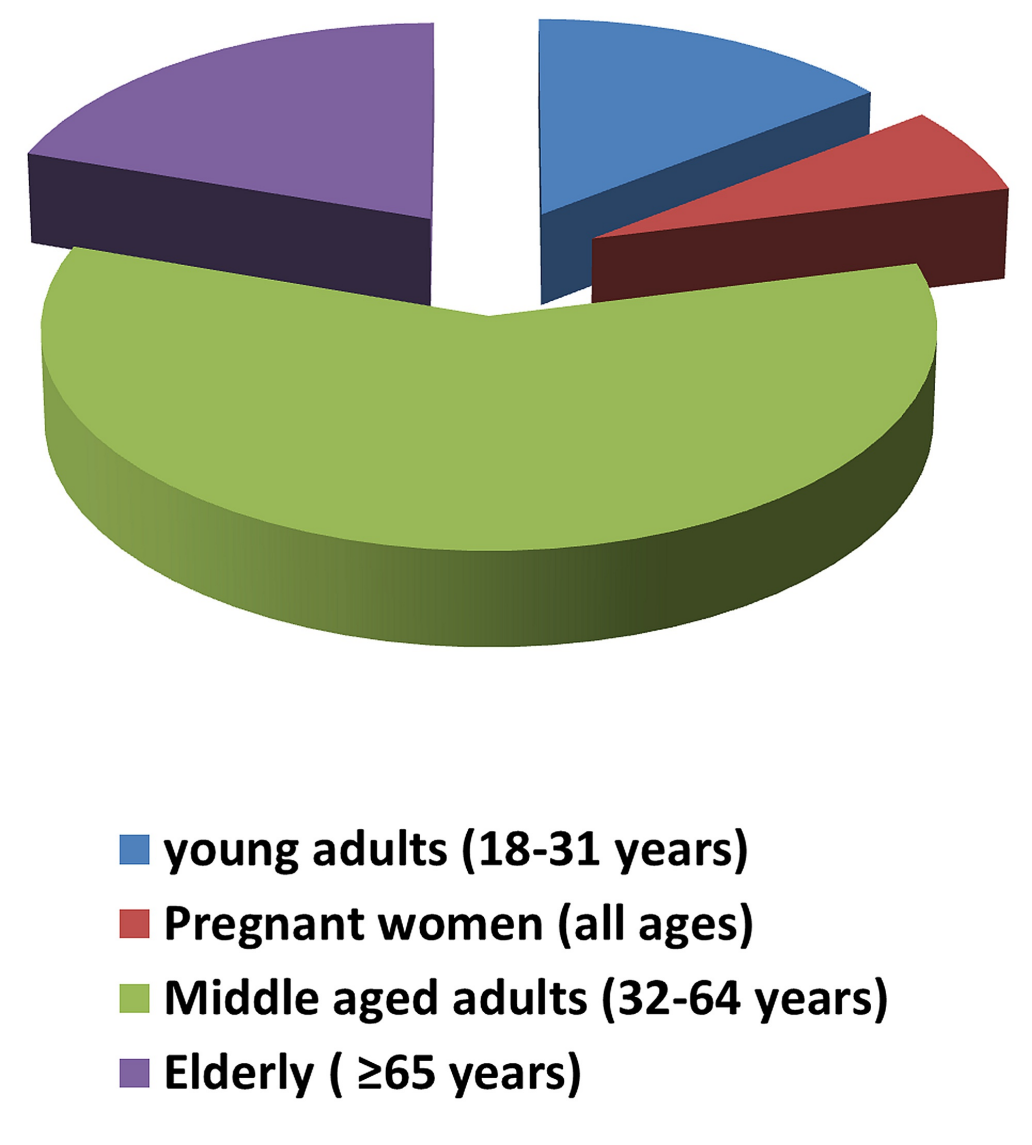
Representation of age distribution of individuals admitted to the hospital with H1N1 infection in 2018

A total of 379 patients were admitted to our hospital in the department of internal medicine with H1N1 infection from January 1, 2018, to December 31, 2018. The median age was 50 years (62-34), out of which there were 75 (19.7%) elderly individuals, 224 (59%) middle-aged adults, and 55 (14.5%) young adults, excluding pregnant women (Table 1, Figure 1). The number of pregnant women included in our study was 25 (6.5%), of which 23 women were in the 18 to 31 age group. Including the pregnant women, the young adults age group represented 20.5% (78) of total admissions while the middle-aged adults group represented 59.6% (226) and the elderly group represented 19.7% (75) of total admissions (Figure 2).

**Table 1:** Age and category distribution IQR: interquartile range

Variable	Numbers (%)	Median age (IQR)
Young adults (18-31 years)	55 (14.5%)	26 (29-24)
Middle-aged adults (32-64 years)	224 (59.1%)	50 (57-42)
Elderly (≥65 years)	75 (19.7%)	69 (67-76)
Pregnant women (18-31 years )	23 (6.0%)	28 (29-26)
Pregnant women (32-64 years)	2 (0.5%)	
TOTAL	379 (100%)	50 (62-34)

Comparison of clinical features

Among the respiratory symptoms seen, cough with expectoration was the most prevalent symptom among young adults, pregnant women, and the elderly group (67.3%, 40%, and 72%, respectively (Table 2). The difference reached statistical significance with more prevalence among the elderly as compared to the other two groups (p-value .013). The elderly group differed from the other two groups in reporting dyspnea (28% vs. 5.5%, 4%), which was highly significant (p=.000) (Table 2). Fever was the most common systemic symptom among all three groups (90.9%, 84%, and 96%). Headache (7.3%, 0%, and 8%) and odynophagia (3.6%, 0%, and 2.7%) were reported less frequently in all three groups, and there was no significant difference between them (Table 2).

**Table 2:** Comparison of clinical features

Prevalent clinical feature	Young adults (N=55), n (%)	Pregnant women (N=25), n (%)	Elderly (N=75), n (%)	Asymp. Sig. (2-sided)
Fever	50(90.)	24 (84)	63 (96)	.206
Headache	4(7.3)	0 (0)	6 (8)	.353
Cough with expectoration	37(67.3)	10 (40)	54 (72)	.013
Dyspnoea	3(5.5)	1 (4)	28 (37.3)	.000
Odynophagia	2 (3.6)	0 (0)	2 (2.7)	.635

Comparison of the prevalence of comorbidities

DM was found in 73.3% of the admitted elderly as compared to 3.6% in young adults and 12% in pregnant women. Hypertension was present in 45% of elderly patients as compared to 1.8% and 4% in young adults and pregnant women, respectively. The difference in prevalence of both DM and hypertension was statistically significant (p=.000) (Table 3). Coronary artery disease (CAD) was significantly (p=.000) prevalent among the elderly at 22.7% as compared to only 1.8% in the young adult group. No instances of CAD were found in pregnant women. CKD was seen only in the elderly group, with a prevalence of 5.3%. Thyroid disease was more prevalent among elderly patients at 20% as compared to 5.5% in young adults and 12% in pregnant women (Table 3). Among the respiratory diseases, the prevalence of bronchial asthma was more or less similar in all groups (9.1% in younger adults, 4% in pregnant women, and 10.7% in the elderly). Chronic obstructive pulmonary disease (COPD) was exclusively seen in elderly patients, at a prevalence of 13.3%. A history of past pulmonary tuberculosis (TB) was present in 2(3.6%) of young adults (Table 3).

**Table 3:** Comparison of the prevalence of comorbidities DM: diabetes mellitus; HTN: hypertension; CAD: coronary artery disease; CKD: chronic kidney disease; BA: bronchial asthma; COPD: chronic obstructive pulmonary disease; TB: tuberculosis a Patient with past history of pulmonary TB b Includes hypothyroidism and hyperthyroidism

Prevalent comorbidity	Young adults (N=55), n (%)	Pregnant women (N=25), n (%)	Elderly (N=75), n (%)	Asymp. Sig. (2-sided)
DM	2 (3.6)	3 (12)	55 (73.3)	.000
HTN	1 (1.8)	1 (4)	34 (45.3)	.000
CAD	1 (1.8)	0 (0)	17 (22.7)	.000
CKD	0 (0)	0 (0)	4 (5.3)	.112
BA	5 (9.1)	1 (4)	8 (10.7)	.602
COPD	0 (0)	0 (0)	10 (13.3)	.003
Past pulmonary TB^a^	2 (3.6)	0 (0)	0 (0)	.159
Thyroid disease^b^	3 (5.5)	3 (12)	15 (20)	.055

The analysis shows that DM, hypertension, COPD, and CAD seem to be significantly prevalent comorbidities among the hospitalized elderly with H1N1 as compared to the other two groups. Other comorbidities, such as CKD, bronchial asthma, thyroid diseases, and past pulmonary TB, are seen to a lesser extent among all three groups, and there was no significant difference in terms of prevalence between them (Table 3).

Comparison of laboratory investigations and chest radiograph

Among the lab investigations, the prevalence of leukopenia was similar in all three groups (13.9% in young adults, 12% in pregnant women, 10.7% in the elderly). However, the prevalence of leukocytosis was significantly higher in pregnant women (32% vs. 26.7% and 7.3%). Lymphopenia was predominant in all three groups, but more frequently seen in pregnant women (76% vs. 61.8% and 41.8%). Lymphocytosis was more prevalent in the young adult group (20% vs. 5.3% and 0%). The majority of patients in all three groups had normal platelet counts. Thrombocytopenia was present in 18.2% of young adults, 4% of pregnant women, and 25.3% of elderly patients. Thrombocytosis was seen in two (3.6%) of the young adults and two (2.7%) of the elderly, but there was no significant difference in the prevalence of thrombocytopenia among all three groups. Elevated creatinine on admission was seen more frequently among the elderly (38% vs. 2% and 0%), which was statistically significant among all three groups (p=.000). See Table 4. Most of these patients subsequently improved within a few days.

**Table 4:** Comparison of laboratory investigations and chest radiograph

Lab features	Young adults (N=55)				Pregnant women (N=25)				Elderly (N=75)				Asymp. Sig. (2-sided)
Total leukocyte count (per mm^3^)	<4500	4500-11,000		> 11,000	<4500	4500-11,000		> 11,000	<4500	4500-11,000		>11,000	.004
Numbers, n (%)	17 (13.9)	34 (61.8)		4 (7.3)	3 (12)	14 (52)		8 (32)	8 (10.7)	47 (62.7)		8 (10.7)	
Lymphocyte percentage in the differential count	<20	20-40		>40	<20	20-40		>40	<20	20-40		>40	.002
Numbers, n (%)	23 (41.8)	21 (38.2)		11 (20)	19 (76)	6 (24)		0 (0)	51 (61.8)	20 (26.7)		4 (5.3)	
Platelet count (lakhs)	<1.5	1.5-4.5		>4.5	<1.5	1.5-4.5		>4.5	<1.5	1.5-4.5		>4.5	.150
Numbers, n (%)	10 (18.2)	43 (78.2)		2 (3.6)	1 (4)	24 (96)		0 (0)	19 (25.3)	54 (72)		2 (2.7)	
Creatinine (mg/dl)	≤1.2		>1.2		≤1.2		>1.2		≤1.2		>1.2		.000
n(%)	53(96.4)		2 (3.6)		25 (100)		0 (0)		54 (72)		21 (38)		
Chest radiograph report, n (%)	Normal		Abnormal		Normal		Abnormal		Normal		Abnormal		.003
	38(69.1)		17 (30.9)		22(88)		3 (12)		39(52)		36 (48)		

An abnormal chest radiograph was reported in a higher number of elderly (48%) than in young adults (30.9%) and pregnant women (12%). This result was statistically significant (p=.003). The abnormalities seen were prominent bronchovascular markings, nonhomogeneous infiltrates, and homogeneous opacification (Table 5).

**Table 5:** Chest radiograph abnormalities

Reported abnormality	Numbers (N=54/155), (34.8%)
Unusually prominent bilateral bronchovascular markings	10
Bilateral non-homogenous infiltrates	12
Left-side non-homogenous opacification	13
Left-side homogenous opacification	9
Right-side non-homogenous opacification	4
Right-side homogenous opacification	6

Hospitalization outcomes

Of the elderly, (26.7%) required more than a week of hospitalization compared to four (7.3%) young adults and four (20%) pregnant women. This result was statistically significant (p=.017). Of the elderly, 24 (32%) required intensive care unit (ICU) admission compared to only one (1.5%) young adult and none of the pregnant women. Ventilator care meant either non-invasive ventilation or invasive ventilation for any duration. More elderly (26.7%) required ventilator support compared to young adults (7.3%) and pregnant women (4%) (Table 6). Bloodstream and urinary tract superinfections were identified only among the elderly group. About 25.3% of these patients had superinfections caused by the organisms outlined in Table 7.

**Table 6:** Hospitalization outcomes compared between the three groups ICU: intensive care unit a Included both non-invasive and invasive ventilation

Outcome variable	Young adults (N=55), n (%)	Pregnant women (N=25), n (%)	Elderly (N=75), n (%)	Asymp. Sig. (2-sided)
Duration of stay (≥7 days)	4 (7.3)	4 (20)	20 (26.7)	.017
Requirement of ventilator support^a^	4 (7.3)	1 (4)	20 (26.7)	.002
Requirement of ICU admission	1 (1.8%)	0 (0)	24 (32)	.000
Superinfections in the blood and urine	0 (0)	0 (0)	19 (25.3)	.000
Death	0 (0)	0 (0)	6 (8)	.036

**Table 7:** Organisms grown from blood and urine cultures of H1N1-infected patients

Organism	Numbers (N=19/155), (12.2%)
Staphylococcus aureus	3
Pseudomonas aeruginosa	5
Enterococcus faecalis	2
Escherichia coli	2
Klebsiella pneumoniae	4
Candida tropicalis	3

Mortality

In the elderly group, there were six (8%) deaths. No deaths occurred in the young adults and pregnant women groups. In terms of hospitalization outcomes, the elderly group had poorer outcomes in all categories. See Table 6

## Discussion

Age and category distribution 

Statistics from the onset of the pandemic in 2009 show that H1N1 predominantly affected young individuals in the age group of 10 to 30 years, constituting nearly 64% of the reported cases while elderly patients only made up about 1.1% [3]. The reason for the over-representation of young adults during the pandemic is postulated to be due to the protection offered by cross-reactive antibodies in elderly people who were born before 1950 from their previous influenza exposures [7,10,16,19]. In our study, the young adult age group, including pregnant women, made up 20.5% of the overall admissions, which is similar to the elderly age group at 19.7%. The middle-aged group formed a major proportion of the representation at 59.6%. This shows an increasing trend of attack rate and hospitalization rate among the older age groups and a gradual decline in the attack rates of young adults compared to the original pandemic data. Pregnant women formed 6.5% of the overall admissions due to H1N1, which is slightly higher as compared to a recent report from China (1.8%) [20] but comparable to a report from the pandemic years by Myles et al. (8%) [6]. Over the past decade, there has been a gradual shift in the age representation of H1N1 infection towards older age groups [8]. In an extensive study done by Dávila et al., a gradual shift in the H1N1 hospitalization rates from young age groups (5-14 years and 15-29 years) towards older age groups (30-44 years and 45-59 years) from 2009 to 2013 was seen [21]. Other authors like Yang et al. [16] and Borja-Aburtoa et al. [13] have also reported a shift in age relative to H1N1 attack rates towards older age groups in the waves following the initial onset of the pandemic. Our study shows a similar age representation, with the attack rate shifting from young adults to the middle-aged and elderly age groups. The most likely explanation for this could be the building immunity among young adults [13,19,21], changes in the circulating strain of the virus [16,19], and inadequate vaccination rates among the middle-aged and elderly population [8,22]. This trend is similar to those of the previous flu pandemics in 1918 and 1968 [19].

Symptomatology

Fever and headache were the most commonly reported systemic symptoms by hospitalized H1N1 infected patients during the pandemic [1,3-4,9]. Cough and dyspnea were the most common respiratory symptoms reported [3-4,9] while odynophagia was reported in varying proportions (82% to 30%) [1,10,22]. Among these symptoms, dyspnea was shown to be associated with intensive care unit (ICU) admission and increased mortality [5-6]. Other symptoms, such as running nose, myalgia, vomiting, and diarrhea, were not analyzed, as they were either inconsistently reported or had lower prevalence [1,3-4,9]. Similar to previous reports, our analysis showed fever and cough to be the most commonly reported symptoms among all three groups. Dyspnea was more prevalent among the elderly group compared with young adults and pregnant women (37.3%, 5.5%, and 4%, respectively), which achieved statistical significance (p<.005). Other symptoms were less frequently reported by all three groups, and there was no significant difference among them. An earlier review suggested the possibility of atypical presentations of H1N1 in elderly patients [7]. However, our analysis showed that elderly people were more symptomatic with respect to respiratory symptoms and reported systemic symptoms similar to young adults. Elderly patients were more likely to report cough with expectoration and breathlessness than young adults and pregnant women while symptomatology-wise, pregnant women were similar to young adults. The increased prevalence of dyspnea in elderly patients correlated with poorer outcomes in terms of ICU admission and mortality, as reported earlier [5-6].

Comorbidities

The comorbidities analyzed in the study were DM, hypertension, bronchial asthma, COPD, CAD, CKD, thyroid diseases, and past pulmonary TB. These comorbidities were chosen based on their commonness in previous prevalence reports [3,5,9-11,23]. They were also associated with poorer outcomes [6,9-10,15,22]. Our study showed a significant (p<.005) prevalence of most comorbidities in elderly patients, including DM, hypertension, CAD, and COPD, as compared to young adults and pregnant women. These comorbidities have been shown to be more prevalent in H1N1-infected elderly patients compared with young adults. In his earlier study including more than 1000 elderly patients, Garnacho-Montero et al. showed that H1N1-infected elderly individuals had more underlying diseases than younger adults [24]. A recent surveillance study conducted from 2015 to 2018 showed that 92.5% of elderly individuals with influenza had at least one comorbidity, among which the most common was cardiovascular disease followed by DM [22]. Our analysis showed a higher prevalence of comorbidities in the hospitalized H1N1-infected elderly that was similar to previous reports, although in our study, DM was the most common comorbidity, followed by hypertension, COPD, and CAD. The comparison shows that comorbidities that are generally prevalent in the elderly are over-represented in elderly patients who are hospitalized with H1N1. This could explain the poorer hospitalization outcomes, including mortality, in this group, as was evident in the analysis.

Laboratory and chest radiograph abnormalities

The common hematological abnormalities reported among H1N1 patients were normal-to-low leukocyte counts and relative lymphopenia [2-3]. The majority of patients (51.8% of young adults, 52% of pregnant women, 62.7% of elderly) in our study had normal leukocyte counts similar to previous reports [2-3,5]. Even though relative lymphopenia was significantly lower (p=0.002) in young adults, it was generally common in all three groups. Earlier reports have shown similar analysis results and, hence, relative lymphopenia could be a good marker of H1N1 infection as compared with other pneumonias [2-3]. We can conclude that relative lymphopenia seems to be more prevalent in H1N1 infection. Many pregnant women had leukocytosis, the reason for which is not clear. Of the elderly patients in the study group, 38% had elevated creatinine on admission as compared to only 3.6% of young adults and none of the pregnant women. Among the elderly who had elevated creatinine on admission, four (5.3%) of them had CKD. In the young adult group, only two (3.6%) patients had elevated creatinine values, which got normalized subsequently. The prevalence of overall renal dysfunction was significantly higher among elderly patients, but the prevalence of CKD on its own was not statistically different from other groups. Chronic and acute renal failure has been shown to be associated with poorer overall outcomes [3,9-10], more so in elderly individuals. Furthermore, acute renal failure has been associated with mortality due to H1N1 [25]. As with previous reports, in our analysis, the elderly group, which had a higher prevalence of renal dysfunction, had poorer overall outcomes.

The prevalence of radiological abnormalities was more common among the elderly group than young adults and pregnant women (48% vs. 12% and 30.9%). Radiologically confirmed pneumonia and an abnormal chest radiograph have been shown to be risk factors for severe outcomes in H1N1 infection [4-6,10,26]. In general, the prevalence of radiological abnormalities varies from 50% to 76% in earlier reports [2,4,26]. Pregnant women did not exhibit a difference in the prevalence or the type of radiological abnormality as compared with their non-pregnant counterparts in an earlier comparison [18]. Contrary to previous studies, radiological abnormalities were less prevalent among the study groups (30.9% of young adults, 12% of pregnant women, 48% of elderly). A possible reason for this could be an earlier presentation of the patients to the hospital or the seasonal variation in the clinical and lab spectrum seen over the multiple waves of H1N1 [8,11,19].

Hospitalization outcomes

In our study, a greater number of elderly patients (26.7%) stayed in the hospital for seven days or more as compared to young adults and pregnant women, which was statistically significant. Being older than 65 years in age seems to be a risk factor for a prolonged duration of hospital stay. The average duration for the hospitalization of influenza patients in the earlier analysis by Arbat et al. was six days [4]. In our study, we divided the duration of stay into categories of less than seven days and seven or more days. The majority of pregnant women (80%) stayed in the hospital for less than a week, which is comparable to results in the study by Suárez-Varela et al., where the majority of pregnant and non-pregnant women stayed in the hospital for less than seven days (75% and 85%, respectively) [18].

There has been a wide variation in the requirement of ICU admission and ventilator support for hospitalized H1N1 patients across various study populations. Overall, ventilator requirements have recently been reported to be 20% by Fu et al. [20] while H Sieh et al. reported a higher rate of ICU admission (44.4%) and ventilator requirement (40.4%) than this [8]. The ICU admission rate and ventilator support requirement have not been well-documented for specific age groups. It is well-known that the majority of patients requiring ventilator support require ICU admission, and the rates are more or less similar [8]. An earlier review showed that pregnant women with H1N1 were 5.8 to 7.4 times more likely to be admitted to the ICU compared to the general population [3]. However, a comparative study between pregnant and non-pregnant women showed ICU admission rates were lower in pregnant women as compared to non-pregnant women (12% and 25.9%, respectively) [18], but this finding was not statistically significant. In our study, none of the pregnant women required ICU admission, and only one required non-invasive ventilation. Though this appears far less, it is comparable with an earlier meta-analysis by Mertz et al. who concluded that pregnancy may not be a risk factor for ICU admission [15]. Of the elderly patients in our study, 32% required ICU admission, which was significantly high when compared with only 1.8% of young adults and 0% of pregnant women requiring the same care. Similarly, 26.7% of elderly patients required ventilator support compared to only 7.3% of young adults and 4% of pregnant women. Both differences were statistically significant (p<.005). The analysis shows that elderly patients are at high risk for ICU admission and ventilator support compared to pregnant women and young adults, as reported in the earlier review by Louie et al., who showed that ICU admission rates were highest in H1N1 patients >50 years of age [27].

In earlier studies, coinfections in H1N1 patients have been shown to contribute to morbidity and mortality, particularly in elderly patients [7]. The majority of studies have been focused on secondary bacterial pneumonias while other major infections (blood, urine) have been ignored [7,10]. Palacios et al. have shown that nasopharyngeal aspirates of nearly 76% of H1N1 patients had a coinfecting organism, out of which only Streptococcus pneumoniae was associated with severe disease [28]. This underlines the importance of bloodstream and other coinfections in H1N1 patients. In this study, we decided to analyze bloodstream and urine coinfections. In the clinical study by Liderot et al., bacterial coinfection was seen in 5.12% of H1N1 patients, out of which, 1.62% were bloodstream infections [29]. In the review by Gilca et al., overall bacterial coinfection in H1N1 patients ranged between 4% to 20% [23]. In our study, bloodstream and urine coinfections were seen in 19 (25.3%) elderly patients. The other two groups did not have evidence of bloodstream or urinary tract coinfections. There was a predominance of gram-negative organisms in the bloodstream infections, along with Staphylococcus aureus, and one patient had candidemia. The spectrum of organisms found in our study is suggestive of hospital-acquired superinfections, rather than community-acquired infections, as was previously seen in elderly patients in the report by Sohn et al. [2]. The prevalence of superinfections in our study is higher, compared to the overall prevalence (1.62%) [29] reported earlier, and the bacterial spectrum is also quite different. The reasons for this are likely the high age-specific prevalence of coinfections in the elderly group because of impaired immunity compared to overall prevalence, and a possible increase in the coinfection rate as compared to previous seasons as reported by Fu et al. They reported an increase in secondary bacterial infections during the 2017-2018 season in H1N1 patients as compared to earlier years (2011-2017; 24.9% and 7.7%, respectively) [20]. It is reasonable to say that elderly patients are more likely to acquire blood- and urine-related hospital-acquired superinfections than young adults and pregnant women. However, more studies are needed to determine the prevalence of hospital-acquired bloodstream infections in H1N1 patients.

The global mortality rate due to H1N1 has varied extensively both regionally and seasonally [12] and has been more prevalent in middle-to-low income countries [1]. Mortality has varied over successive years and seasons due to antigenic drift. In 2019, Nawal et al. reported the circulating strain A/Michigan/45/2015, which has replaced the strain A/California/7/2009 in India and has been associated with slightly lower mortality rates but has significantly affected the population with more comorbidities [25]. During the 2009 H1N1 pandemic, attack rates and relative risk of death were higher for younger adults [3,14] and children, whereas the elderly population was relatively immune due to previous influenza exposures in agreement with the antigen sin hypothesis [3,19]. Although fewer elderly individuals were infected during the pandemic, proportion-wise, mortality was substantially higher in this demography as compared to younger individuals [7,23]. Defective innate and adaptive immunity to new antigens and underlying chronic diseases could contribute to this disparity in mortality rate [7]. There has been an age shift in attack rates and mortality rates from the younger age group to the older age groups over the waves and seasons following the 2009 pandemic, as reported by Borja-Aburtoa et al. [13] and Yang et al. [16]. The reasons for this are likely related to the build-up of immunity and improved vaccination rates in younger adults [13,16,21]. A report by Hsieh et al. during the 2013-2014 season showing increased attack rate and mortality in middle-aged adults 50-64 years of age rather than young adults further supports this fact [8]. Our study aligns with the above evidence that the mortality rate has shifted from the younger age group to the older age groups. Our study showed 8% mortality in the elderly group and no mortality in the younger adult group. Pregnancy has been considered to be an established risk factor for hospitalization and poor hospital outcomes related to H1N1 infection [3,9-10,17]. The suppression of cellular immunity and the elevation of humoral immunity, associated with hormonal and physiological changes in the body, are the proposed reasons for this [17]. Pregnant women are at a 4.3 to 7 times higher risk for hospitalization, a 5.8 to 7.4 times higher risk for ICU admission, and a 10 times higher risk of death [3]. In contrast, our study did not show pregnancy as a risk factor for poor hospitalization outcomes. Many authors have made similar conclusions previously. In the meta-analysis conducted by Duggal et al. on global mortality, overall mortality was 31% in critically ill adult patients as compared to critically ill pregnant women (10%) [12]. In their cohort study on predictors for clinical outcomes in H1N1, Myles et al. concluded that pregnancy was not significantly associated with poorer outcomes [6] while there was an increased risk for mortality at an age >50 years instead. In a direct comparison, Suárez-Varela et al. found that pregnant women were not at an increased risk of hospitalization or complications as compared with non-pregnant women [18]. In the meta-analysis by Mertz et al., which included 234 articles, elderly patients had higher rates of hospitalization and a significant risk of death as compared to the younger population due to H1N1 (odds ratio: 2.95). They also concluded that pregnancy was not associated with an increased risk of death, though it was associated with increased rates of hospitalization [15]. Similarly, in the meta-analysis by Tricco et al., it was suggested that the possibility of ethnicity and comorbidities increased the risk of hospitalization in pregnant women, rather than pregnancy itself. In the same report, elderly patients were found to be at a higher risk for hospitalization and death [30]. Gilca et al. also found that ages >60 years was a risk factor for mortality in H1N1 while pregnancy was not associated with an increased risk of mortality [23].

Our study supports the fact that old age is a risk factor for an increased rate of hospitalization, especially in the case of more comorbidities and coinfections, and results in an increased risk of poorer hospital outcomes such as prolonged stay, need for ICU admission or ventilator support, and death. On the contrary, our study does not show pregnancy and young age to be independent risk factors for all categories of poorer hospital outcomes. The reasons for this might be the build-up of immunity in the younger population, including pregnant women [13,16,21]; the change in virulence pattern and attack rates of the circulating H1N1 strain [16,21,25]; established variation in attack rates and mortality of influenza over multiple waves and seasons as noted by various authors [6,13,16,19,21]; and possible improving, yet insufficient, vaccination rates in pregnant women and young adults. Elderly people continue to be at risk of poorer outcomes, which is likely due to the increased prevalence of comorbidities [24], possible delays in presentation and treatment [24], immune senescence [7], and poor vaccination rates [7,22].

Limitations

Our study has several limitations. First, the study is based on a hospitalized population in a single year. The population studied might have included a small symptomatic group with significant illness while leaving out a major group of less symptomatic or asymptomatic patients in the community, leading to possible selection bias. Second, the statistics might have been affected by the seasonal variability that is seen in H1N1 infection over the years. Hence, the generalization of the results might require more longitudinal studies. Third, prior vaccination history, which might influence hospitalization outcomes, was not analyzed in the study population. Fourth, in the pregnant group, gestational age was not analyzed, as few earlier reports suggested that gestational age might play a role in the prognosis of H1N1-infected pregnant women. And finally, since the study was aimed at a comparison between three specific groups, the major demographic of middle-aged patients was not included in the analysis while pediatric patients were excluded. Hence, overall hospitalization outcomes could not be analyzed.

## Conclusions

Our study shows that age representation in hospitalization due to H1N1 seems to have shifted from younger to older age groups over the past decade, which is similar to previous pandemics. Elderly patients were at a greater risk for prolonged hospital stays, ICU admission, the need for ventilator support, and death as compared to young adults and pregnant women. Pregnancy and young adulthood may not be associated with poor hospitalization outcomes for H1N1, as has been earlier thought. The age shift, build-up of immunity, and changing virulence patterns might be the reasons for this association. This study highlights the need for improving vaccination rates and healthcare strategies in elderly individuals in regards to influenza. More longitudinal studies are needed to evaluate pregnancy as a risk factor in H1N1 outcomes and hospital-acquired superinfections in H1N1-infected patients.
